# Novel mechanisms underlying HSV‐1‐driven neurodegeneration

**DOI:** 10.1002/alz70855_102922

**Published:** 2025-12-23

**Authors:** Giovanna De Chiara, Virginia Protto, Mariya Timotey MITEVA, Francesco Zanzi, Maria Elena Marcocci, Domenica Donatella Li Puma, Francesco Pastore, Cristian Ripoli, Roberto Piacentini, Claudio Grassi, Anna Teresa Palamara

**Affiliations:** ^1^ Institute of Translational Pharmacology, CNR, Rome, Rome, Italy; ^2^ Istituto Superiore di Sanità, Rome, Rome, Italy; ^3^ University of Rome Sapienza, Rome, Rome, Italy; ^4^ Università Cattolica del Sacro Cuore, Rome, Rome, Italy; ^5^ Fondazione Policlinico Universitario A. Gemelli IRCCS, Rome, Italy; ^6^ Università Cattolica del Sacro Cuore, Rome, Italy; ^7^ Istituto Superiore di Sanità, Rome, Italy

## Abstract

**Background:**

Recurrent herpes simplex virus type‐1 (HSV‐1) infection has been repeatedly proposed as a potential risk factor for Alzheimer's disease (AD). Our previous studies have provided evidence that HSV‐1 promotes the accumulation of AD‐specific biomarkers, including amyloid beta and hyperphosphorylated tau proteins (ptau), neuroinflammation, and cognitive deficits. Recent research has suggested novel mechanisms involved in AD pathogenesis: extracellular vesicles (EVs), which are key players in cell‐to‐cell communication, facilitate the dissemination of neurotoxic proteins, including ptau, among neuronal cells; the aberrant upregulation of the complement cascade, a critical component of the innate immune system, contributes to synaptic elimination in the AD brain. Here, we investigate whether HSV‐1 infection/reactivation activates these pathways in *in vivo* and *ex vivo* models.

**Method:**

Molecular, biochemical, virological, and functional analyses were performed on in vitro (primary cultures of murine neurons and co‐cultures with microglial BV2 cells), in vivo (wild‐type BALB/c mice), and ex vivo (hippocampal organotypic brain slices) models of HSV‐1 infection.

**Result:**

We found that HSV‐1 exploits EVs to disseminate ptau among brain cells in in vitro and in vivo infection models. Importantly, the HSV‐1‐induced EV‐bearing ptau can be undertaken by recipient neurons, thus likely contributing to misfolding and aggregation of native tau, as reported for other AD models. We also revealed that HSV‐1 infection causes a significant increase in complement proteins at both mRNA and protein levels and their release in the supernatant of infected cells. Interestingly, we detected the complement components C1q and C4 in synaptosomes purified from HSV‐1‐infected neurons, suggesting they may concur in HSV‐1‐driven synaptic damage. Moreover, HSV‐1 infection in co‐cultures of brain cells triggers an increase in microglial phagocytosis of synaptic material, which was partially prevented when the complement cascade was inhibited by neutralizing anti‐C3 antibody or the C3 convertase inhibitor compstatin. Most of these data were confirmed in HSV‐1‐infected organotypic hippocampal slices. Importantly, here we detected a significant decrease in spine density that was rescued when the infection was performed in the presence of complement inhibitors.

**Conclusion:**

Overall, our data highlight several mechanisms contributing to HSV‐1‐induced neurodegeneration.

